# Oncoplastic Surgery and the Clinical Features of Breast Cancer—Relevant Factors Associated with Reoperation in Breast Oncoplastic Surgery

**DOI:** 10.3390/jcm11030817

**Published:** 2022-02-03

**Authors:** Alicja Forma, Robert Sitarz, Jacek Baj, Krzysztof Sołowiej, Sergiusz Łukasiewicz, Andrzej Stanisławek

**Affiliations:** 1 Department of Forensic Medicine, Medical University of Lublin, 20-090 Lublin, Poland; aforma@onet.pl; 2 Department of Human Anatomy, Medical University of Lublin, 20-090 Lublin, Poland; jacekbaj@umlub.pl; 3 Department of Surgical Oncology, Center of Oncology of the Lublin Region St. Jana z Dukli, 20-091 Lublin, Poland; ksolowiej@cozl.pl (K.S.); snakef630@gmail.com (S.Ł.); stanlub@mp.pl (A.S.); 4 Department of Oncology, Oncology and Environmental Health, Medical University of Lublin, 20-090 Lublin, Poland

**Keywords:** breast cancer, breast-conserving surgery, treatment, reoperation, oncoplastic surgery, oncoplasty

## Abstract

Oncoplastic breast surgery slowly becomes a part of routine breast cancer surgical management but evidence with regard to oncological safety remains limited. The aim of this study was to compare relevant factors associated with the particular type of breast carcinoma and the applied surgical techniques either with or without oncoplastic surgery. This retrospective study enrolled the breast cancer female patients who underwent breast-conserving therapy alone or with the oncoplastic surgery in the Department of Surgical Oncology at the Center of Oncology of the Lublin Region St. Jana from Dukli in the years 2008–2011. The study involves 679 breast cancer patients who underwent oncoplasty (*n* = 81) and the control group (*n* = 598). There is a significant relationship between the histological type of breast cancer (*p* = 0.00000) along with the expression of estrogen and/or progesterone receptors (*p* = 0.01285) and the usage of oncoplastic surgery in breast cancer patients. Interestingly, in the majority of cases, there was no need to conduct a reoperation. Oncoplastic surgery is an effective and safe strategy that might be favorable especially for those patients who are potential candidates for more invasive surgical methods. High-quality evidence to support the oncological safety and benefits of oncoplastic breast surgery is lacking.

## 1. Introduction

Breast cancer is the most prevalent cancer among females, being responsible for more than 1.5 million new diagnoses per year, and the incidence rate is still increasing [1]. At the same time, breast cancer is considered to be the second leading cause of cancer-related deaths in women [2]. The classification of both the histological and molecular types of breast cancer depends on several pathological and clinical features. The major histological classification includes such types as invasive ductal carcinoma (the highest prevalence rate) [3], lobular carcinoma, medullary carcinoma, cribriform carcinoma, mucinous carcinoma, oncocytic carcinoma, neuroendocrine carcinoma, or apocrine carcinoma, while the number is continually increasing because of different combinations of the pathological features shared between the above-mentioned conditions [4]. Molecular breast cancer subtypes include the HER2/NEU, triple-negative, luminal B, and luminal A, with the latter likely being the most prevalent [5].

Currently, there are several treatment strategies as well as surgical techniques for breast cancer patients, and the choice of the most convenient method primarily depends on the size and location of the tumor; however, other aspects such as the size of the breast or the personal wishes of the patient are also taken into consideration. The most common types of breast cancer surgery include breast-conserving therapy (BCT) and mastectomy which are both well-established therapies for breast cancer patients [6]. Even though several breast-conserving surgical procedures such as partial mastectomy, tumorectomy, or lumpectomy seem to be beneficial for patients due to their limited and effective surgical approach, the aesthetic values remain unsatisfactory to some extent.

Recently, the period of convalescence after the operation of breast cancer patients along with the aesthetic values have been significantly improved due to advancements in the therapeutic strategies and the surgical techniques more often chosen by the surgeons. Regarding the aesthetic outcomes, oncoplastic surgery seems to be one of the most promising methods, providing an opportunity to improve breast cancer patients’ satisfaction after the operation along with the health-related quality of life and so-called ‘physical well-being’ [7,8]. Oncoplastic surgery combines the removal of breast cancer tumor with reconstructive surgery at the same time often with surgery of the opposite breast providing more satisfactory cosmetic outcomes for patients.

In this retrospective cohort study, we aimed to compare the histology of breast tumor, removal of the local lymph nodes, TNM classification, expression of the estrogen (ERs), progesterone (PRs), and HER2 receptors, Bloom–Richardson–Elston (BRE) grading system, and reoperation after the oncoplastic surgery or other BCT techniques.

## 2. Patients and Methods

**Figure 1:**
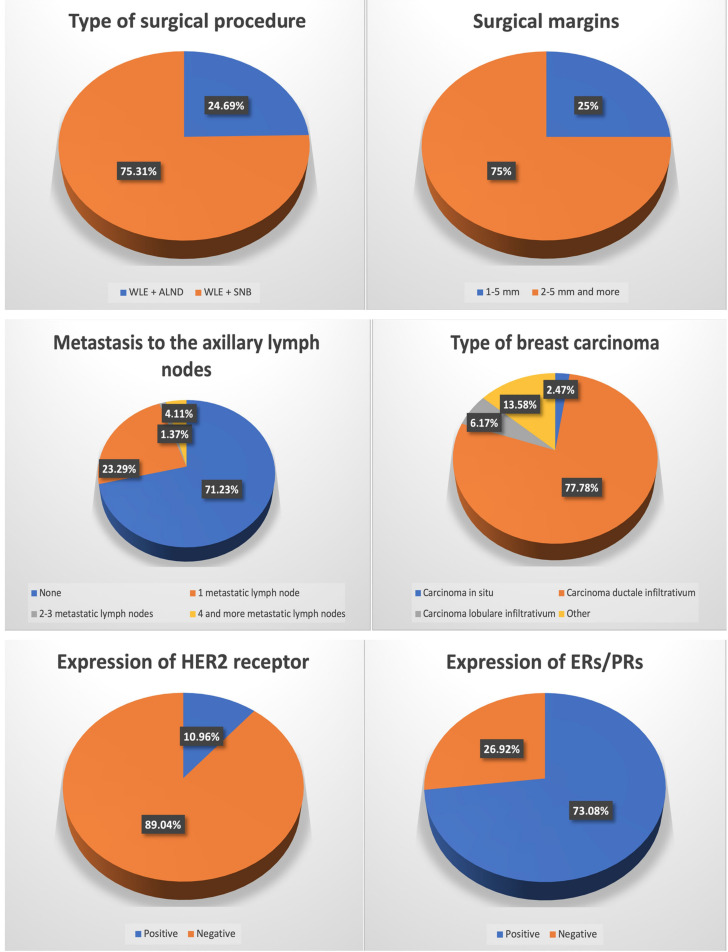

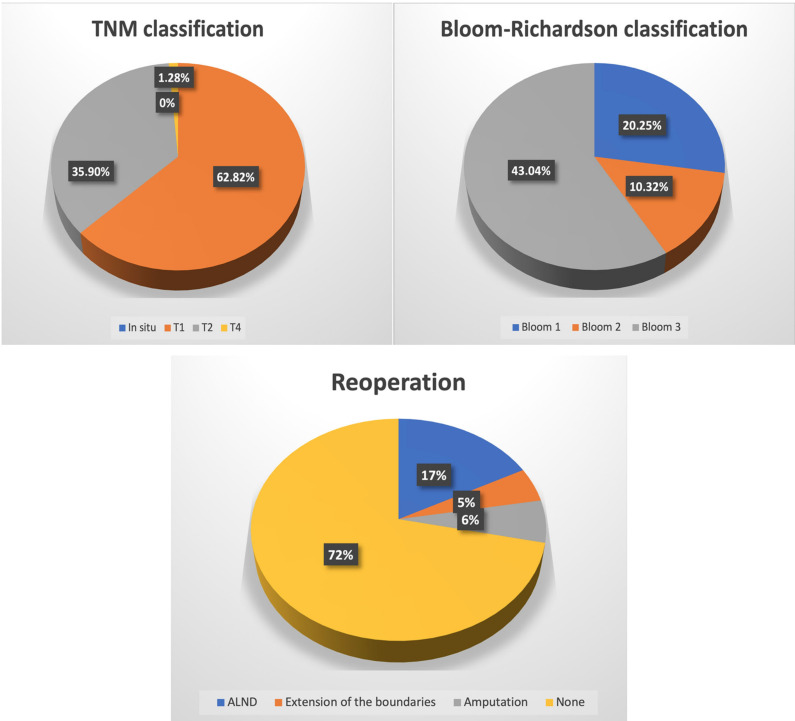
Characteristics of breast cancer patients who underwent oncoplastic surgery.

This retrospective cohort study enrolled the breast cancer patients who underwent BCT alone or with the oncoplastic surgery in the Department of Surgical Oncology at the Center of Oncology of the Lublin Region St. Jana from Dukli in the years 2008–2011. The total number of 679 female patients was included in this study and further categorized into two groups—the control group without oncoplastic surgery conducted (*n* = 598) and the study group with oncoplasty (*n* = 81) (Figure 1). Each patient was diagnosed based on World Health Organization criteria, and tumor differentiation grade was classified according to Edmondson and Steiner. Liver function was evaluated using the Child–Pugh scoring system. Tumor stages were classified based on the International Union against Cancer TNM classification system. Just after the surgery and 6 months after the surgery, all the patients in both—the control and study group—along with the doctors, were asked to fulfill the assessment of the outcomes of the operation including such information as the shape, nipple position, volume, visible scars, symmetry, patient’s satisfaction, and overall aesthetic outcome.

The mean age of patients in the study group was 56.54 ± 9.47, whereas in the control group, the mean age was 54.92 ± 9.68. Primary surgical techniques included (1) wide local excision (WLE) with sentinel lymph node biopsy (SNB) or (2) BCT (WLE with axillary lymph node dissection (ALND)). Both of the above-mentioned techniques were used in the study group as well as in the control group. Surgical procedures were performed by the experienced breast surgeon, who chose the most suitable surgical procedure in a particular situation based on the size and location of the breast tumor, which was all in agreement with the patients’ will. All of the histopathological analyses that aimed to assess the histological type of breast tumor were performed by the experienced pathologist specializing in breast pathologies. The following types of carcinomas have been categorized in this study: carcinoma in situ, carcinoma ductale infiltrativum, carcinoma lobulare infiltrativum, and less prevalent carcinomas such as gelatinosum, tubulare, medullare, apocrinale, mucionsum, microcellulare, macrocellulare, papilare infiltrativum, cribiforme infiltrativum, phylloides malignus, according to the classification criteria (2013) [9]. The criteria of the International Union against Cancer TNM classification system were applied to classify the tumor stages. Reoperation was categorized into three major groups depending on the surgical technique used—(1) ALND, (2) extension of the borders, and (3) mastectomy. All of the information regarding the type of surgical procedure, surgical margins, histological type of breast carcinoma, metastases to the axillary lymph nodes, TNM classification, surgical intervention in the lymph system, expression or ERs, PRs or HER2 receptors, Bloom–Richardson classification, and reoperation and surgical technique used were collected from the electronic patient records. The characteristics of the study and control groups are presented in Table 1. This study was approved by the Local Committee on Medical Ethics of the Medical University of Lublin (KE-0254/53/2021) and was performed in compliance with national legislation and the Declaration of Helsinki.

## 3. Statistical Analysis

Statistical analyses between the study and control group were calculated using the χ^2^ test and those included the following variables—the histological type of carcinoma, removal of the metastatic lymph nodes, TNM classification, expression of ERs, PRs, and HER2 receptors, Bloom–Richardson classification, and reoperation. Then, we have conducted a multivariate regression analysis along with the calculation of the odds ratio (OR) with the confidence intervals using StatSoft, Poland Statistica v. 10.0 software. A *p*-value was considered to be statistically significant when it was less than 0.05.

## 4. Results

The characteristics of the breast cancer patients along with the results of the χ^2^ test are summarized in Table 2.

In both of the groups, WLE + SNB was more prevalently chosen compared to BCT (WLE + ALND). In the study and control groups, the surgeon decided to establish the surgical margins greater than 2 mm (2–5 and more) in most of the cases—*n* = 60 and *n* = 437, respectively. In the study group, *carcinoma ductale infiltrativum* was the most prevalent (*n* = 63; 77.78%), while *carcinoma* in situ (*n* = 2; 2.47%) was the least. A similar prevalence was noted in the case of the control group, except that *carcinoma* in situ was the least prevalently diagnosed similarly to *carcinoma lobulare infiltrativum—n* = 29; 4.85% and *n* = 29; 4.85%, respectively. In most of the cases, there was no metastasis to the local lymph nodes in the study group (*n* = 52; 71.23%), nor in the control group (*n* = 423; 72.68%). In the study group, metastases to 2–3 lymph nodes were the least prevalent (*n* = 1; 1.37%), whereas in the control group, four and more metastatic lymph nodes were the least prevalent (*n* = 34; 5.84%). According to the TNM classification, T1 was the most prevalent in both of the groups (*n* = 49; 60.50% and *n* = 384; 64.21%, respectively), while the least prevalent was T4—*n* = 1; 1.23% and *n* = 6; 1.00% for study and control groups, respectively. Regarding the intervention in the lymph system, ALND was more prevalently chosen than SNB in both of the groups. In most of the cases, HER2 expression was negative whereas the expression of either ERs or PRs or both was positive in both groups. In the study group, Bloom 3 was most prevalent (*n* = 34; 43.04%) while in the control group it was Bloom 2 (*n* = 252; 89.68%). In both of the groups, most frequently there was no reoperation performed, and whenever there was a need to perform it, ALND was the most prevalently performed one—*n* = 14; 17.28% and *n* = 62; 10.37% in a study and control group, respectively (Table 3).

There is a significant relationship between the undertaken oncoplastic surgery and the histological type of breast cancer. Either in case of *carcinoma ductale infiltrativum* or other invasive types of carcinoma (except for *ductale*/*lobulare infiltrativum carcinoma* but including *carcinoma* in situ, *carcinoma ductale infiltrativum*, *carcinoma lobulare infiltrativum*, *carcinoma*; *gelatinosum*, *tubulare*, *medullare*, *apocrinale*, *mucionsum*, *microcellulare*, *macrocellulare*, *papilare infiltrativum*, *cribiforme infiltrativum*, *phylloides malignus*) there was a statistical relationship between the above-mentioned histological type and the oncoplastic surgery (*p* = 0.00000 and *p* = 0.00000, respectively). However, there was no statistical relationship between the oncoplastic surgery performed in the case of patients with *carcinoma lobulare infiltrativum* (*p* = 0.26437). In addition, breast cancer patients who underwent oncoplastic surgery tend to express either ERs or PGs or both of the receptors (*p* = 0.01285), while there was no statistical relationship in the case of HER2 receptor expression (*p* = 0.55891). There were no statistical differences regarding the reoperation rates, removal of the lymph nodes, as well as TNM and Bloom–Richardson classifications in none of the groups while applying the χ^2^ test.

The results obtained with the usage of the multivariate regression analysis have been separated into four groups taking into account the relationship between the oncoplastic surgery and (1) clinical features of breast carcinoma, (2) the applied treatment, (3) reoperation, and (4) the number of the metastatic lymph nodes removed. The above-mentioned results are presented in Table 4, Table 5, Table 6 and Table 7.

Regarding the clinical features of breast carcinoma, the multivariate regression analysis showed that there was a significant relationship between the performed oncoplastic surgery and the histological type of carcinoma which was *carcinoma ductale infiltrativum* (*p* = 0.005) (OR = 0.13, 95% CL 0.03–0.54). There was no statistical difference in terms of other histological types of breast carcinoma and the undertaken oncoplastic surgery. Likewise, there was no difference in cases when ALND (*p* = 0.069), (OR = 0.56 (95% CL 0.30–1.05)) was applied in the study group. However, when ALND was applied along with the reoperation, the statistical significance has been observed in this group of those patients who underwent oncoplastic surgery (*p* = 0.045), (OR = 1.94, 95% CL 1.01–3.72). There was no statistical difference in the case of the number of the metastatic lymph nodes removed, except for the case when four or more nodes were removed with ALN (*p* = 0.030), (OR = 0.10, 95% CL 0.01–0.80). We asked the patients who underwent radiotherapy to fulfill the subjective scale of outcomes just before and after 6 months after radiotherapy. The results are in Table A1 and Table A2. Further, we also asked the doctors to assess the outcomes of the patients who underwent radiotherapy. The results are presented in Table A3 and Table A4. Doctors and patients who were allocated to the control group were also asked to assess the outcomes of the operation just after the surgery and after 6 months as well. The results are presented in Table A5, Table A6, Table A7 and Table A8.

Regarding the follow-up patient care, during the first 2 years after the operation, the patients were obliged to visit the doctor every 3 months; during the next 3 years, every 6 months; and then, patients needed to visit the doctor once a year for the control. Every year after the operation, each patient had mammograms, chest X-rays, as well as abdomen ultrasound scans (USG) performed. Other tests and examinations were performed according to the patients’ individual needs according to their symptoms.

## 5. Discussion

Patients with breast cancer should receive the most appropriate type of treatment, taking into account the characteristics of carcinoma as well as any concomitant comorbidities. Until the development of oncoplasty, the limited choice of surgical procedures that mainly came down to radical mastectomy or segmental excision with radiotherapy remained a significant limitation for both the patients and surgeons. Currently, approximately two-thirds of breast cancer patients receive conventional BCT, while according to the recent data, the safety and effectiveness of oncoplastic surgeries remain at a similar level with additional advantages such as better cosmetic outcomes [10]. Recently, more widely applied techniques of plastic surgery enabled a satisfactory tumor excision as well as a reduction in potential deformities associated with the surgery. Oncoplastic surgery constitutes a surgical approach for tumors that are unfavorably localized enabling the minimization of unsatisfactory cosmetic defects at the same time being safe for breast cancer patients as it combines the oncologic and reconstructive surgery at the same time. Even though oncoplastic surgery is a relatively new course of treatment, some modifications of this method such as autologous free dermal fat graft are emerging [11]. Compared to other BCT such as wide local excision, lumpectomy or quadrantectomy, oncoplastic surgery allows for the removal of larger tumors, which in consequence provides lower re-excision rates [12,13,14,15]. What is crucial is the fact that usually during oncoplasty, the surgical margins might be more easily increased compared to other BCT without drastic aesthetic outcomes; however, it is recommended to avoid wide margins in both the oncoplastic surgery and other BCT [16,17,18,19]. Regarding the aesthetic outcomes, patients who underwent oncoplastic surgery tend to present excellent or very good outcomes, even though in many cases, most of the patients present with larger and multifocal tumors, proving the utility and effectiveness of oncoplasty at the same time [11,20]. The aesthetic aspects include the preservation of the most satisfactory breast symmetry along with adequate reconstruction despite the breast size. The application of oncoplastic surgery enables an effective excision of tumors even up to four times more extensive in volume compared to the traditional tumorectomy [16]. The results of the oncoplastic surgery performed are considered to be more satisfactory for patients than BCT or mastectomy [21]. What is intriguing is that scars that remain after oncoplastic surgery tend to be larger compared to those after alternative surgeries; however, due to post-operative irradiation, the overall outcome is more satisfactory for patients [22]. While performing oncoplastic surgery, the surgeons should be aware of the potential side effects and complications such as glandular necrosis, surgical site infections, or delayed wound healing; however, with the current state of knowledge, those are equivalent to or even less prevalent compared to other breast surgery methods [21,23]. Moreover, patients who receive neoadjuvant chemotherapy are not at greater risk of surgery-related complications, making this procedure even safer [10]. Our study consisted of 679 breast cancer patients among which only 81 had oncoplastic surgery performed. The results of our study showed that there is a statistical significance between the performed oncoplastic surgery and the histological type of cancer which was either *carcinoma ductale infiltrativum* (*p* = 0.00000) or other types of invasive cancer described in this study except for *carcinoma lobulare infiltrativum* (*p* = 0.00000). Oncoplastic surgery was also performed more prevalently among patients with carcinoma characterized by the expression of ERs or PRs (*p* = 0.01285). Moreover, oncoplastic surgery was significantly associated with the type of reoperation which was ALND (*p* = 0.045), and removal of equal or more lymph nodes (*p* = 0.030) compared to the classic surgery. In most of the cases in our study, there was no need for reoperation either in the study or in the control group. This outcome is similar to data described in the literature stating that the rate of tumor recurrence after oncoplastic surgery is very low and varies between 0% and 2% [24,25,26]. In our study, patients who underwent oncoplastic surgery presented good short-term outcomes, similar to the group with other BCT. This data corresponds to other research that also presented highly satisfactory short- along with long-term outcomes after oncoplasty [27]. This data is in compliance with other studies that indicate that similarly to other BCT, oncoplastic surgeries are characterized by safety and similar or even lowered need for further reoperations [28,29].

## 6. Conclusions

Our study indicates that the application of oncoplastic surgery seems to be an effective and safe breast-conserving strategy in patients with breast carcinoma. Oncoplastic surgery appears to be safe as other breast-conserving surgery/therapy which seems to be beneficial, especially for patients who were potential candidates for more invasive surgical strategies such as mastectomy. Further, our retrospective study suggests that the involvement of oncoplastic surgery is associated with great satisfaction of the patients along with good aesthetic outcomes. Therefore, it can be assumed that such good results might affect the postoperative quality of life of patients in a significant manner compared to the patients who do not undergo oncoplastic surgery.

## Figures and Tables

**Table 1:** Characteristics of the study and control groups.

Characteristics		Study Group (81 Patients)	Control Group (598 Patients)
Type of surgical procedure	BCT (WLE + ALND)	20 (24.69%)	145 (24.25%)
	WLE + SNB	61 (75.31%)	453 (75.75%)
Surgical margins	1 to 5 mm	20 (25%)	153 (25.93%)
	2–5 mm and >5 mm	60 (75%)	437 (74.07%)
Type of breast carcinoma	Carcinoma in situ	2 (2.47%)	29 (4.85%)
	*Carcinoma ductale infiltrativum*	63 (77.78%)	465 (77.76%)
	*Carcinoma lobulare infiltrativum*	5 (6.17%)	29 (4.85%)
	Other *	11 (13.58%)	74 (12.37%)
Metastasis to the axillary lymph nodes	None	52 (71.23%)	423 (72.68%)
	1 metastatic lymph node	17 (23.29%)	86 (14.78%)
	2–3 metastatic lymph nodes	1 (1.37%)	39 (6.7%)
	4 and more metastatic lymph nodes	3 (4.11%)	34 (5.84%)
TNM classification	In situ	0 (0%)	8 (1.34%)
	T1	49 (60.50%)	384 (64.21%)
	T2	28 (34.57%)	153 (25.59%)
	T3	3 (3.70%)	47 (7.86%)
	T4	1 (1.23%)	6 (1.00%)
Surgical intervention in the lymph system	SNB	13 (16.05%)	152 (25.42%)
	ALND	68 (83.95%)	446 (74.58%)
Expression of ERs and/or PRs **	Positive	57 (73.08%)	475 (84.37%)
	Negative	21 (26.92%)	88 (15.63%)
Expression of HER2 receptor	Positive	8 (10.96%)	73 (13.42%)
	Negative	65 (89.04%)	471 (86.58%)
Bloom–Richardson classification	Bloom 1	16 (20.25%)	105 (18.72%)
	Bloom 2	29 (10.32%)	252 (89.68%)
	Bloom 3	34 (43.04%)	204 (36.36%)

* Including carcinomas: gelatinosum, tubulare, medullare, apocrinale, mucionsum, microcellulare, macrocellulare, papilare infiltrativum, cribiforme infiltrativum, phylloides malignus; ** expression of either ERs or PRs or both.

**Table 2:** Characteristics of the breast cancer patients with the results of the χ^2^ test.

		Study Group *n* = 81		Control Group *n* = 598		*p*-Value
		*n*	%	*n*	%	
Histological type of breast cancer	*Carcinoma ductale infiltrativum*	38	46.91	490	81.94	0.00000
	Other	43	53.09	108	18.06	
Histological type of breast cancer	*Carcinoma lobulare infiltrativum*	2	2.47	32	5.35	0.26437
	Other	79	97.53	566	94.65	
Histological type of breast cancer	Other invasive than *carcinoma ductale*/ *lobulare infiltrativum* *	36	44.44	50	8.36	0.0000
	Other	45	55.56	548	91.64	
Removal of 1 lymph node	Positive metastasis	22	28.57	158	27.34	0.81950
	Negative metastasis	55	71.43	420	72.66	
Removal of 2 ≥ positive metastatic lymph nodes	ALN (1–2 and more metastatic lymph nodes)	10	12.99	67	11.59	0.72101
	Other (1≤)	67	87.01	511	88.41	
Removal of 4 ≥ positive metastatic lymph nodes	ALN (3 ≤ metastatic lymph nodes)	76	98.7	542	93.77	0.07838
	ALN (4 ≥ metastatic lymph nodes)	1	1.3	36	6.23	
TNM classification	Tis	2	2.86	6	1.07	0.20928
	Other	68	97.14	553	98.93	
TNM classification	T1	49	70	384	68.69	0.82401
	Other	21	30	175	31.31	
TNM classification	T1 + T2	68	97.14	546	97.67	0.78348
	Other	2	2.86	13	2.33	
Expression of ERs and PRs	Positive **	57	73.08	475	84.37	0.01285
	Negative ***	21	26.92	88	15.63	
Expression of HER2 receptor	Positive	8	10.96	73	13.42	0.55891
	Negative	65	89.04	471	86.58	
Bloom–Richardson classification	Bloom = 1	16	20.25	105	18.72	0.74400
	Other	63	79.75	456	81.28	
Bloom–Richardson classification	Bloom = 1 + 2	45	56.96	357	63.64	0.25048
	Bloom = 3	34	43.04	204	36.36	
Reoperation—ALND	Performed	14	17.28	62	10.44	0.06746
	None	67	82.72	532	89.56	
Reoperation—extension of the boundaries	Performed	4	4.94	39	6.57	0.57372
	None	77	95.06	555	93.43	
Reoperation—mastectomy	Performed	5	6.17	52	8.75	0.43316
	None	76	93.83	542	91.25	

* carcinoma in situ, *carcinoma ductale infiltrativum*, *carcinoma lobulare infiltrativum*, *carcinoma—gelatinosum*, *tubulare*, *medullare*, *apocrinale*, *mucionsum*, *microcellulare*, *macrocellulare*, *papilare infiltrativum*, *cribiforme infiltrativum*, *phylloides malignus*. ** Expression of at least one type of receptor—either ERs or PRs; *** None of the receptors is expressed; Abbreviations: ALN—axillary lymph node, ERs—estrogen receptors, PRs—progesterone receptors.

**Table 3:** Prevalence and types of surgical methods used during reoperation.

Reoperation	Study Group (81 Patients)	Control Group (598 Patients)
ALND	14 (17.28%)	62 (10.37%)
Extension of the borders	4 (4.94%)	39 (6.52%)
Mastectomy	5 (6.17%)	52 (8.70%)
None	58 (71.61%)	445 (74.41%)

**Table 4:** The multivariate regression analysis on the performed oncoplastic surgery and the clinical features of the breast carcinoma.

	*p*-Value	OR	No. Patients Who Underwent Oncoplastic Surgery	−95% CL	+95% CL	1/OR
T: T1, T2 vs. T3, T4	0.358	0.36	68 (11.1%) vs. 2 (13.3%)	0.04	3.17	2.76
Expression of ERs and PRs: positive vs. negative	0.185	0.60	57 (10.7%) vs. 21 (19.3%)	0.28	1.28	1.67
Expression of HER2: positive vs. negative	0.312	0.61	8 (9.9%) vs. 65 (12.1%)	0.23	1.59	1.64
ALN: positive vs. negative	0.696	0.87	22 (12.2%) vs. 55 (11.6%)	0.42	1.77	1.15
Histopathology: *carcinoma ductale infiltrativum* vs. other	0.005	0.13	38 (7.2%) vs. 43 (28.5%)	0.03	0.54	7.74
Histopathology: *carcinoma lobulare infiltrativum* vs. other	0.097	0.18	2 (5.29%) vs. 79 (12.25%)	0.02	1.37	5.66
Histopathology: invasive carcinoma other than *carcinoma ductale infiltrativum* and *carcinoma lobulare infiltrativum* vs. other	0.456	1.73	36 (41.9%) vs. 45 (7.6%)	0.41	7.34	0.58
Bloom: 3 vs. 1.2	0.920	1.03	34 (14.4%) vs. 45 (11.2%)	0.55	1.94	0.97

**Table 5:** The multivariate regression analysis on the performed oncoplastic surgery and the applied treatment.

	*p*-Value	OR	No. Patients Who Underwent Oncoplastic Surgery	−95% CL	+95% CL	1/OR
ALND: performed vs. not performed	0.069	0.56	13 (7.9%) vs. 68 (13.2%)	0.30	1.05	1.78

**Table 6:** The multivariate regression analysis on the performed oncoplastic surgery and the reoperation.

	*p*-Value	OR	No. Patients Who Underwent Oncoplastic Surgery	−95% CL	+95% CL	1/OR
ALND: performed vs. not performed	0.045	1.94	14 (18.4%) vs. 67 (11.2%)	1.01	3.72	0.51
Extension of the borders: performed vs. not performed	0.562	0.73	4 (9.3%) vs. 77 (12.2%)	0.25	2.11	1.37
Mastectomy: performed vs. not performed	0.252	0.57	5 (8.8%) vs. 76 (12.3%)	0.21	1.50	1.77

**Table 7:** The multivariate regression analysis on the performed oncoplastic surgery and the number of the metastatic lymph nodes removed.

	*p*-Value	OR	No. Patients Who Underwent Oncoplastic Surgery	−95% CL	+95% CL	1/OR
ALN: positive vs. negative	0.979	1.01	22 (12.2%) vs. 55 (11.6%)	0.60	1.68	0.99
Removal: 2 or more	0.083	2.20	10 (12.9%) vs. 67 (11.6%)	0.90	5.38	0.45
Removal: 4 or more	0.030	0.10	1 (2.7%) vs. 76 (12.3%)	0.01	0.80	10.45

## Data Availability

Not applicable.

## Abbreviations

ALND	axillary lymph node dissection
BCT	breast-conserving therapy
ERs	estrogen receptors
OR	odds ratio
PRs	progesterone receptors
SNB	sentinel lymph node biopsy
WHO	World Health Organization
WLE	wide local excision

## Appendix A

**Table A1:** Subjective scale of the outcomes of the operation completed by the patients just before radiotherapy.

The Assessed Characteristic	Number of Patients			
Shape	3	8	42	25
Nipple position	4	10	37	30
Volume	8	10	43	20
Visible scars	10	11	34	26
Symmetry	9	23	29	20
General satisfaction	7	17	38	19
Grading scale	1	2	3	4

Grading scale 1–4: 1—very poor result; 2—poor result; 3—good result; 4—excellent result.

**Table A2:** Subjective scale of the outcomes of the operation completed by the patients 6 months after radiotherapy.

The Assessed Characteristic	Number of Patients			
Shape	6	11	36	19
Nipple position	5	14	39	23
Volume	9	12	41	19
Visible scars	9	13	35	24
Symmetry	12	27	26	16
General satisfaction	9	20	34	18
Grading scale	1	2	3	4

Grading scale 1–4: 1—very poor result; 2—poor result; 3—good result; 4—excellent result.

**Table A3:** The assessment of the outcomes of the operation completed by the doctors just before radiotherapy.

The Assessed Characteristic	Number of Patients			
Shape	4	11	45	21
Nipple position	3	12	41	25
Volume	10	16	33	22
Visible scars	7	10	44	20
Symmetry	14	22	26	19
Overall aesthetic outcome	5	24	37	15
Grading scale	1	2	3	4

Grading scale 1–4: 1—very poor result; 2—poor result; 3—good result; 4—excellent result.

**Table A4:** The assessment of the outcomes of the operation completed by the doctors 6 months after radiotherapy.

The Assessed Characteristic	Number of Patients			
Shape	7	15	41	18
Nipple position	3	17	39	22
Volume	10	17	34	21
Visible scars	4	12	44	21
Symmetry	14	24	27	16
Overall aesthetic outcome	9	21	38	13
Grading scale	1	2	3	4

Grading scale 1–4: 1—very poor result; 2—poor result; 3—good result; 4—excellent result.

**Table A5:** Subjective scale of the outcomes of the operation completed by the patients allocated in the control group just after the surgery.

The Assessed Characteristic	Number of Patients			
Shape	93	154	246	105
Nipple position	89	146	268	95
Volume	100	179	289	30
Visible scars	78	212	217	91
Symmetry	68	197	208	125
Satisfaction	61	167	231	139
Grading scale	1	2	3	4

Grading scale 1–4: 1—very poor result; 2—poor result; 3—good result; 4—excellent result.

**Table A6:** Subjective scale of the outcomes of the operation completed by the patients allocated in the control group 6 months after the surgery.

The Assessed Characteristic	Number of Patients			
Shape	119	215	185	79
Nipple position	114	221	189	74
Volume	132	156	277	33
Visible scars	70	204	240	84
Symmetry	103	248	160	87
Satisfaction	89	210	198	101
Grading scale	1	2	3	4

Grading scale 1–4: 1—very poor result; 2—poor result; 3—good result; 4—excellent result.

**Table A7:** The assessment of the outcomes of the operation completed by the doctors just before the surgery of the patients allocated in the control group.

The Assessed Characteristic	Number of Patients			
Shape	100	171	253	74
Nipple position	76	179	197	146
Volume	112	193	214	79
Visible scars	97	306	119	76
Symmetry	98	179	245	76
Overall aesthetic outcome	79	193	228	98
Grading scale	1	2	3	4

Grading scale 1–4: 1—very poor result; 2—poor result; 3—good result; 4—excellent result.

**Table A8:** The assessment of the outcomes of the operation completed by the doctors 6 months after surgery of the patients allocated in the control group.

The Assessed Characteristic	Number of Patients			
Shape	124	204	222	48
Nipple position	114	205	243	36
Volume	103	198	227	70
Visible scars	79	243	207	69
Symmetry	114	207	219	58
Overall aesthetic outcome	96	235	186	81
Grading scale	1	2	3	4

Grading scale 1–4: 1—very poor result; 2—poor result; 3—good result; 4—excellent result.
